# Dendritic cell biology and its role in tumor immunotherapy

**DOI:** 10.1186/s13045-020-00939-6

**Published:** 2020-08-03

**Authors:** Yingying Wang, Ying Xiang, Victoria W. Xin, Xian-Wang Wang, Xiao-Chun Peng, Xiao-Qin Liu, Dong Wang, Na Li, Jun-Ting Cheng, Yan-Ning Lyv, Shu-Zhong Cui, Zhaowu Ma, Qing Zhang, Hong-Wu Xin

**Affiliations:** 1 State Key Laboratory of Respiratory Disease, Affiliated Cancer Hospital & Institute of Guangzhou Medical University, Guangzhou, 510095 China; 2 Laboratory of Oncology, Center for Molecular Medicine, School of Basic Medicine, Faculty of Medicine, Yangtze University, 1 Nanhuan Road, Jingzhou, 434023 Hubei China; 3 Department of Biochemistry and Molecular Biology, School of Basic Medicine, Faculty of Medicine, Yangtze University, Jingzhou, 434023 Hubei China; 4 Department of Gynaecology, Comprehensive Cancer Center, Hannover Medical School, 30625 Hannover, Germany; 5 Stanford University, Stanford, CA 94305 USA; 6 Department of Laboratory Medicine, School of Basic Medicine, Faculty of Medicine, Yangtze University, 1 Nanhuan Road, Jingzhou, 434023 Hubei China; 7 Department of Pathophysiology, School of Basic Medicine, Faculty of Medicine, Yangtze University, Jingzhou, 434023 Hubei China; 8 Department of Medical Imaging, School of Basic Medicine, Faculty of Medicine, Yangtze University, Jingzhou, 434023 Hubei China; 9 Department of Oncology, First Affiliated Hospital of Yangtze University, Jingzhou, Hubei China; 10 Institute for Infectious Diseases and Endemic Diseases Prevention and Control, Beijing Center for Diseases Prevention and Control, Beijing, 100013 China; 11 State Key Laboratory of Biocontrol, School of Life Sciences, Sun Yat-sen University, Guangzhou, 510275 China; 12 Institute of Sun Yat-sen University in Shenzhen, Shenzhen, China; 13 People’s Hospital of Lianjiang, Lianjiang, 524400 Guangdong China

**Keywords:** Dendritic cells (DCs), MHC, Immune cells, Tumor immunotherapy

## Abstract

As crucial antigen presenting cells, dendritic cells (DCs) play a vital role in tumor immunotherapy. Taking into account the many recent advances in DC biology, we discuss how DCs (1) recognize pathogenic antigens with pattern recognition receptors through specific phagocytosis and through non-specific micropinocytosis, (2) process antigens into small peptides with proper sizes and sequences, and (3) present MHC-peptides to CD4^+^ and CD8^+^ T cells to initiate immune responses against invading microbes and aberrant host cells. During anti-tumor immune responses, DC-derived exosomes were discovered to participate in antigen presentation. T cell microvillar dynamics and TCR conformational changes were demonstrated upon DC antigen presentation. Caspase-11-driven hyperactive DCs were recently reported to convert effectors into memory T cells. DCs were also reported to crosstalk with NK cells. Additionally, DCs are the most important sentinel cells for immune surveillance in the tumor microenvironment. Alongside DC biology, we review the latest developments for DC-based tumor immunotherapy in preclinical studies and clinical trials. Personalized DC vaccine-induced T cell immunity, which targets tumor-specific antigens, has been demonstrated to be a promising form of tumor immunotherapy in patients with melanoma. Importantly, allogeneic-IgG-loaded and HLA-restricted neoantigen DC vaccines were discovered to have robust anti-tumor effects in mice. Our comprehensive review of DC biology and its role in tumor immunotherapy aids in the understanding of DCs as the mentors of T cells and as novel tumor immunotherapy cells with immense potential.

## Introduction

Antigen presenting cells (APCs) play a significant role in both innate and adapted immunity responses. The category of APCs consists of macrophages, dendritic cells (DCs), and B lymphocytes [1]. DCs, first discovered by Ralph Steinman in 1973, are the most important of the APCs and have many different subtypes. These subtypes have a variety of special functions in immunological processes, such as initiating immune reactions, regulating immune responses, and maintaining those responses [2]. According to its ontogeny, a DC can be categorized as either a conventional DC (cDC) or a plasmacytoid DC (pDC), as summarized in Table 1 [3]. Also, according to the developmental stage of the DC, it can be classified into two major categories: immature and mature [4]. Most immature DCs reside on mucosal surfaces, with the skin and solid organs acting as sentinels to recognize antigens. These DCs have a lower expression of major histocompatibility complex (MHC) I and MHC II, T cell co-stimulation factors, and adhesion molecules [3]. Immature DCs do not secrete proinflammatory cytokines. However, they are capable of migration.

**Table 1:** DC classification

DC subtype	Identification basis	Presence in vivo	Main surface markers		Secreted molecules	Function
			Mouse	Human		
pDCs	120G8+, B220+, CD11c+, LY6C+, CD11b–	Circulate through the blood and lymphoid tissues	TLR7, TLR9, TLR12, RLR, STING, CLEC12A	TLR7, TLR9, RLR, STING, CLEC12A	CD317, SIGLECH, B220, BDC2*, BDC4*	(1) Type I interferons, (2) antigen presentation, (3) T cell priming
cDC1s	cDC1s (XCR1hiCD172low)	Thymus, spleen and lymph nodes	TLR2-, TLR4, TLR11–TLR13, STING, CLEC12A	TLR1, TLR3, TLR6, TLR8, TLR10, STING, CLEC12A	XCR1, CLEC9A, (CD103), (CD8α), BDCA3*	Cross-priming
cDC2s	cDC2s (XCR1lowCD172hi)	Thymus, spleen and lymph nodes	TLR1, TLR2, TLR4–TLR9, TLR13, RLR, NLR, STING, CLEC4A, CLEC6A, CLEC7A, (CLEC12A)	TLR1–TLR9, RLR, NLR, STING, LEC4A, CLEC6A, CLEC7A, CLEC10A, CLEC12A	CD11b, SIRPa, (CD4), (DCIR2)	CD4+ T cell priming
MoDCs	CD11c+, Ly6C+, CD103	Differentiate from monocytes in peripheral tissues on inflammation. Resident in skin, lung, and intestine	CD11c+, MHC-II+, CD11b+, Ly6C+, CD64+, CD206+, CD209+, CD14+, CCR2+	CD11c+, MHC-II+, CD11b+, Ly6C+, CD64+, CD206+, CD209+, CD14+, CCR2+, CD103+	CD11b, CCR2, LY6C, CD115	Inflammation

**Table 2:** Fc receptor classification and function

FcR	Type	Affinity of binding IgG	Function domain	Fc signal	Fc expression cells		Short-term effects	Long-term effects
					Constitutive	Inducible		
FcR I	I	High	Fc domains within IgG	ITAM	Monocytes	Neutrophils, eosinophils, dendritic cells	—	—
FcrRIIa	I	Low	Fc domains within IgG	ITAM	Monocytes, neutrophils, eosinophils, macrophages, dendritic cells, platelets, granulocytes	—	Degranulation, ROI production, phagocytosis, cytokine, chemokine expression, platelet activation	Proinflammatory molecule stimulation and release, cell survival, motility, platelet binding to leukocytes, enhanced antigen process, presentation, T cell responses
FcrRIIb	I	Low	Fc domains within IgG	ITIM	B cells, monocytes, neutrophils, eosinophils, macrophages, dendritic cells, plasma cells	—	B cell selection	High-affinity IgG responses
FcrRIIIa	I	Low	Fc domains within IgG	ITAM	NK cells	Dendritic cells	Phagocytosis, cytokine and chemokine expression, cell activation, degranulation	Monocyte recruitment, differentiation, proinflammatory pathway stimulate, cytotoxicity, cell survival, effector leukocyte impact, immune complexes generation
FcrRIIIb	I	Low	Fc domains within IgG	ITAM	Granulocytes	Neutrophils	Degranulation, ROI production, phagocytosis	Proinflammatory molecule release, cell survival, motility, myeloid leukocyte impact
DC-SIGN	II	—	Sialylated Fc glycoforms				Phagocytosis, cytokine and chemokine expression	Macrophage polarization, IgG-mediated inflammation
DC23	II	—	Sialylated Fc glycoforms		B cells	T cells, monocytes, neutrophils, eosinophils	B cell selection	Constitutive high-affinity IgG responses

Note: High-affinity FcγR, capable of binding monomeric IgG; low-affinity FcγR, variable affinities by subclasses

When immature DCs uptake antigens, they shift to secondary lymphoid organs and present antigens to helper T cells or effector T cells to trigger specific cytotoxic T lymphocyte (CTL) responses [5]. In the meantime, they also gradually become more motile and upregulate the expression of CC-chemokine receptors 7, 8 (CCR7, 8) [6].

On the other hand, matured DCs have a reduced ability to uptake and process antigens but have an enhanced migration capacity. In addition, mature DCs were also reported to have an increased expression of various co-stimulatory molecules—for instance, CD40, CD70, and CD80, as well as CD86—and an increased production of proinflammatory cytokines and chemokines [7, 8].

Here, we review the latest studies on DCs as the mentors of T cells, with an emphasis on how DCs specifically recognize, process, and present antigens to program T cells for immune activation, suppression, or memorization. We also highlight some recent developments that demonstrate the immense potential of DCs in tumor immunotherapy.

## Antigen recognition and internalization

**Fig. 1:**
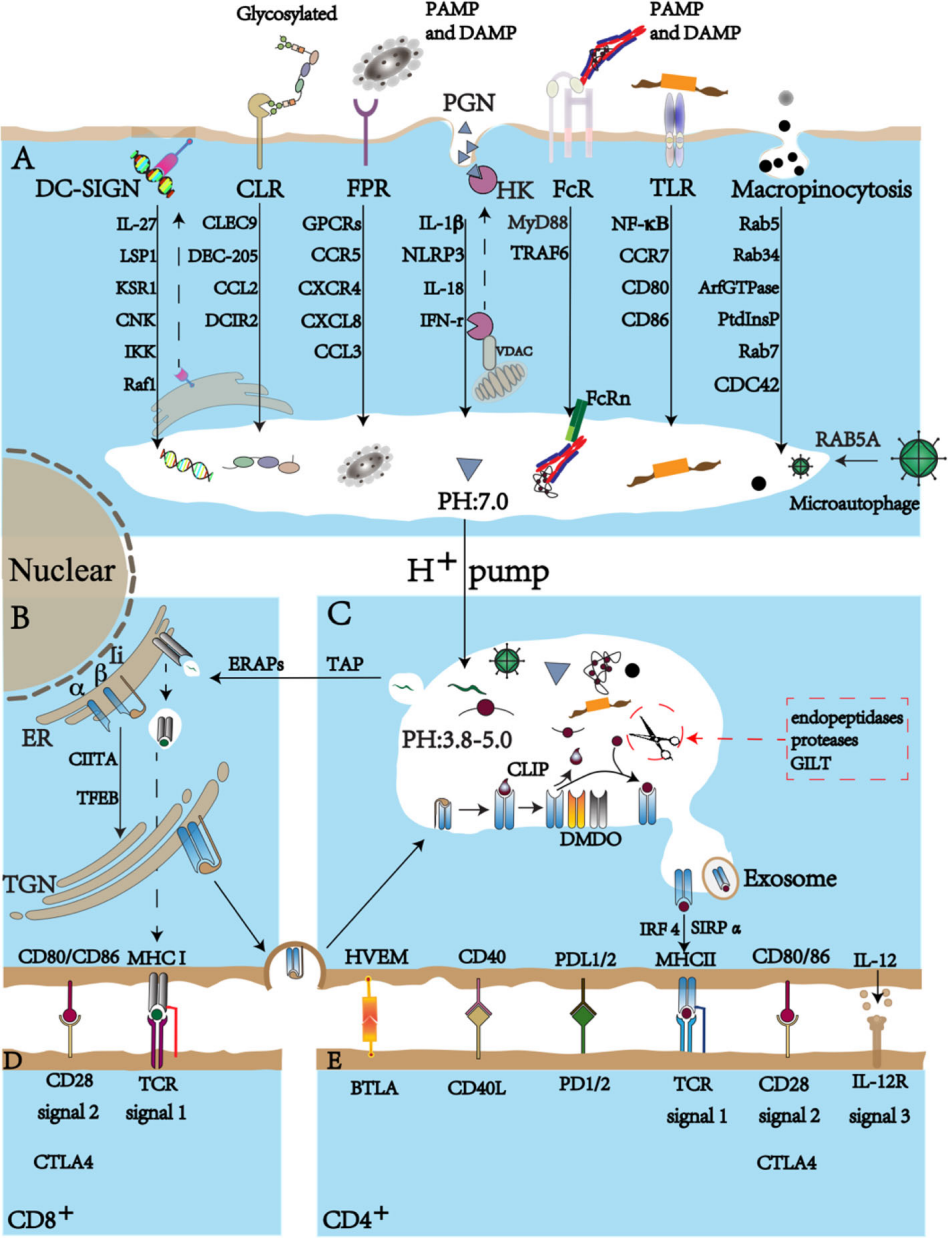
Pathways of antigen recognition, processing, and presentation of DCs. **a** Antigen recognition and internalization into the early endosome through specific phagocytosis (microautophagy and chaperone-mediated autophagy) or non-specific macropinocytosis. **b** Dimers of MHC-I and MHC-II are formed in the endoplasmic reticulum (ER). MHC-II binds with a non-polymorphic invariant chain Ii (CD 74). **c** Gradual acidification to approximate pH 3.8–5.0 by the ATP-dependent vacuolar proton pump, increasing the lysosomal enzyme activity in the late endosomal and lysosomal-processing compartments. After proteolytic cleavage, antigens are transferred to MHC molecules. **d** MHC-I antigen cross-presentation involved in modulating receptor-mediated signaling. **e** MHC-II antigen presentation involved in modulating receptor-mediated signaling

DCs are highly dynamic, using their specific receptors to recognize foreign invading antigens or aberrant self-antigens. DCs recognize antigens through pathogen-associated molecular patterns (PAMPs) as well as danger-associated molecular patterns (DAMPs) through pattern recognition receptors (PRRs). DCs then uptake, process, and present antigens to T cells to initiate immune responses (Fig. 1a).

### Specific phagocytosis

The critical approach of antigen uptake by DCs and other immune cells is generally believed to be phagocytosis [7]. There are two important forms of phagocytosis: microautophagy and chaperone-mediated autophagy (Fig. 1a). Microautophagy is initiated when the expression of master regulator RAB5A is altered and the MHC-II compartment (MIIC) is fused by the autophagy protein LC3. As a key endocytic protein, the master regulator RAB5A also has multiple physiological activities, such as promoting coherent and ballistic collective motility, impacting junctional mechanics and monolayer rigidity, and increasing endomembrane trafficking [9]. Chaperones, such as C-type lectins and Fc receptors, can recognize antigens by targeting special ligands of apoptotic cells or pathogens. Afterwards, the endocytosis process, mediated by clathrin, is induced, which places antigens into antigen-processing compartments [10]. Here, we have highlighted the major mechanisms of chaperone-mediated autophagy.

#### C-type lectin receptors (CLRs)

Pattern-recognition receptors are critical components to immune responses. They recognize invading microbes and induce protective immune responses to infection. CLRs, a type of pattern-recognition receptor, are central to antifungal immunity. They are expressed on macrophages as well as DCs. Dectin-1, CLEC9, and DEC-205 (lymphocyte antigen 75) are all examples of CLRs [10]. Specifically, the calcium-dependent carbohydrate recognition domains (CRD) in CLRs recognize conserved fungal cell-wall carbohydrates and their glycosylation pattern, also known as the carbohydrate fingerprint [11]. The melanin-sensing C-type lectin receptor MelLec plays a major role in antifungal immunity by recognizing naphthalene-diol of 1,8-dihydroxynaphthalene (DHN)-melanin. MelLec has the ability to identify the conidial spores of Aspergillus fumigatus and other DHN-melanized fungi [12, 13]. The C-Type lectin 5A (CLEC5A) is a spleen tyrosine kinase (Syk)-coupled receptor of APC and plays a pivotal role in the activation of innate immunity against viruses—especially *Flavivirus* [14]. CLEC5A promotes neutrophil extracellular trap development and the production of both reactive oxygen species and proinflammatory cytokines by recognizing the bacteria *Listeria monocytogenes*. It can also induce inflammasome activation in macrophages and stimulate the immune reaction of T cells [14].

The human DC-specific intercellular adhesion molecule-1 grabbing nonintegrin (DC-SIGN or CD209) is thought to be a canonical of the C-type lectin receptor expressed on both macrophages and DCs [15]. It is a type 2, mannose-specific C-type lectin that also works as a cytosolic DNA-sensor. It induces specific immune responses upon the recognition of glycans through its carbohydrate recognition domains (CRD) [16, 17]. After DC-SIGN recognizes fucose-based PAMPs, it activates IKKε. In turn, IL-27 is produced, follicular T helper cell (TFH) differentiation is facilitated, B cell IgG production is stimulated, B cell survival is aided, and Th2 differentiation is implemented [18, 19]. DC-SIGN can be bound by adaptor protein LSP1 in combination with a triad “signalosome” complex consisting of the adaptor proteins KSR1, CNK, and kinase [19]. The binding of pathogens to these lectins results in an internalization to endosomal compartments, where the pathogens are destroyed and an immune response is initiated [16]. In addition, Chao et al. found that Annexin A2 (ANXA2), which is abundantly expressed in nasopharyngeal carcinoma (NPC), can activate DC-SIGN and inhibit DC-mediated immunity against NPC [20]. Both DC maturation and the production of proinflammatory interleukin (IL)-12 were inhibited, but the production of immunosuppressive IL-10 was increased [20].

#### Formyl peptide receptors (FPRs)

FPRs are G protein-coupled receptors (GPCRs) expressed in bone marrow cells and especially on DCs [21]. GPCRs belong to the group of pattern-recognition receptors that can recognize peptides containing N-formylated methionine [21]. There are three human FPRs: FPR1, FPR2, and FPR3. The mice equivalents are unclear [22]. FPRs can induce DC migration to necrotic tumor cells and affect tumor angiogenesis [23]. They can also downregulate the cell surface expression of GPCRs, CCR5, CXCR4, chemokines CXCL8 (also referenced as interleukin 8, IL-8), and CCL3, which in turn promotes monocyte migration, which is involved in tumor growth [24, 25]. FPRs have five antigen-binding pockets where consecutive amino acid residues can be modified without changing their affinity towards the agonists [26]. FPRs can also induce cell adhesion with the robust release of migrating superoxide granules by recognizing transducing chemotactic signals in phagocytes [26]. Yousif et al. reported that the recognition of the uPAR (84-95) sequence and the shorter synthetic peptide (Ser88-Arg-Ser-Arg-Tyr92, SRSRY) was a fresh, powerful, and steady repressor against FPR1-triggered monocyte trafficking and cell migration [26].

#### NOD-like receptor NLRP3 and Hexokinases

The glycolytic enzyme hexokinase is an innate immune receptor which monitors bacterial peptidoglycan (PGN) by recognizing PGN-produced N-acetylglucosamine (NAG) in the cytosol [27]. The degradation of Gram-positive bacterial cell walls by the phagosomes of DCs will lead to the activation of the NOD-like receptor family, pyrin domain-containing 3 (NLRP3), which promotes the release of hexokinase [27]. Moreover, when NAG binds with hexokinase, it induces the secretion of proinflammatory interleukins IL-1β and IL-18 [27, 28]. Uncontrolled IL-1β release can lead to autoinflammatory diseases such as Cryopyrin-associated periodic syndrome (CAPS) or Mediterranean fever. The overproduction of IL-18 can also cause autoinflammatory diseases such as rheumatoid arthritis. IL-18 functions to promote inflammation primarily through stimulating the production of IFN-γ, which is a classic anti-microbial inflammatory cytokine [29].

#### Fc receptors

Expressed on hematopoietic cells, Fc receptors (FcRs) play an important role in immune responses by binding to the Fc region of an antibody. FcRs can bind to different immunoglobulins (IgA, IgM, IgE, and IgG), participating in antibody-mediated innate and adaptive immune responses (Table 2) [30]. A review by van de Winkel has introduced the classification of Fc receptors in detail [31]. In humans, activated Fc receptors include FcRI (CD64), FcRIIA (CD32a), FcRIIC (CD32c), FcRIIIA (CD16a), and FcRIIIB (CD16b) [32]. Most members of the Fc receptor family generally bind to extracellular IgGs, excluding the neonatal Fc receptor (FcRn) and the intracellular Fc acceptor tripartite motif-containing protein 21 (TRIM21). FcRI has the highest affinity for monomeric IgG1, the lowest affinity for monomeric IgG2, and a medium level of affinity for IgG3 and IgG4. Mostly, FcRI is saturated and in a steady condition in the presence of physiological serum. The binding complexes (FcR-IgG) not only trigger activating signals, but also mediate inhibitory signals [33]. The complexes affect the intensity of the immune reactions by setting-up a “threshold” via a tyrosine-based activation motif (ITAM) or immune receptor tyrosine-based inhibitory motif (ITIM) in their cytoplasmic tails. ITIM phosphorylation has an immunosuppressive effect by inducing the recruitment of phosphatases, including SHIP-1 and inositol polyphosphate-5-phosphatase (INPP5D). Recent studies suggest that only monocyte-derived DCs and macrophages express high levels of activated Fc receptors for IgG [34]. FcRn works as an intracellular IgG Fc binding receptor and is encoded by the Fcgrt gene. FcRn is a lifelong resident of the endolysosomal system in most hematopoietic cells, including DCs, and can guide antibody-bound viruses and other antigens to the proteasome by activating E3 ubiquitin ligase [33]. After the FcRs-IgG-peptide complex internalization is completed via FcRn, FcRn releases IgG-peptides into the acidifying endosomes, where the peptides can be successfully processed into peptide epitopes to be loaded onto MHC-I or MHC-II molecules to activate CD8^+^ or CD4^+^ T cells [35, 36].

FcRn in DCs can also lead directly to the activation of CD4^+^ T cells [37]. An experiment showed that DCs isolated from wild type mice pre-incubated with IgG-peptides were able to effectively prime CD4^+^ T cells [37]. In contrast, DCs isolated from Fcgrt^−/−^ mice needed antigen concentrations nearly 1000 times higher than that for normal mice, suggesting that FcRn significantly strengthens the ability of DCs to generate MHC-II compatible epitopes from antigens delivered by IgG-peptides [38].

#### Toll-like receptors (TLRs)

Discovered in 1996, TLRs are type I transmembrane proteins [39]. TLRs reside on the surfaces of immune cells or intracellular compartments and recognize PAMPs for immune responses against pathogens and neoplastic cells. TLRs induce DC maturation by activating nuclear factor kappa B (NF-κB) and upregulating the expression of CCR7, MHC-II, and co-stimulatory CD80 or CD86 [40, 41]. At least two members of the Toll-like receptor (TLR) family—TLR7 and TLR9—can recognize self-RNA/DNA, respectively [42]. A new report found that the TLR trafficking chaperone UNC93B1 specifically limited the signaling of TLR7, but not TLR9, and prevented TLR7-dependent autoimmunity in mice [42]. Comprehensive analyses reveal that both TLR2 and TLR4 are required to recognize Sel1, activate NF-κB and MAPK signaling pathways, and lead to the expression of proinflammatory cytokines and chemokines against *Candida albicans* infections [43].

TLRs are also expressed on tumor cells for the purpose of immune evasion [44]. The stimulation of TLR3 and TLR5 signaling can induce an anti-tumor T cell response. However, TLR4, TLR7, TLR8, and TLR9 mediated chronic inflammations were found to have pro-tumor effects. On the other hand, a novel PAMP-mimicking regent can activate macrophage-mediated tumor immunotherapy.

A specific agonist of TLR2 modified by acetyl groups with a substitution degree of 1.8 (acGM-1.8) was found to stimulate macrophages to release anti-tumor proinflammatory cytokines. Another small-molecule agonist of TLR7, 2-methoxyethoxy-8-oxo-9-(4-carboxybenzyl) adenine (1V209), was found to enhance adjuvant activity and limit adverse events when conjugated to hollow silica nanoshell s[45].

### Non-specific macropinocytosis

Macropinocytosis is a type of non-specific phagocytosis in the form of cell “drinking”. It can be spontaneously induced by the engagement of growth factors, chemokines, or Toll-like receptors (TLRs) [11, 46]. TLRs are dependent on extracellular Ca2^+^-sensing receptors (CaSR) [47]. Regulatory factors like Rab5, Rab34, and ArfGTPases contribute to early macropinosome maturation [12]. Rab5 and PtdIns (3)P then synergize to promote fusion with early endosomes with the involvement of EEA1 [10]. The homotypic fusion and protein sorting (HOPS) complex, septins, and SNARE proteins endow the late compartment vacuoles with vacuolar-type H^+^-ATPase (V-ATPase) at low pH values so that degradative enzymes can function optimally [13]. At this moment, a critical switch from Rab5 into Rab7 promotes the centripetal transportation of the vacuole and its fusion with late endosomal/lysosomal compartments.

## MHC expression, assembly, and trafficking in DCs

MHC molecules have two categories: MHC class I (MHC-I) and MHC class II (MHC-II) [48]. They both exhibit tremendous allelic polymorphism in the peptide-binding groove. This allows them to bind with a diverse range of peptides (Fig. 1b).

### MHC expression

MHC class I molecules are heterodimers that consist of two polypeptide chains: α and β2-microglobulin (B2M). The two chains are linked noncovalently via the interaction of B2M and the α3 domain. Only the α chain is polymorphic and encoded by a HLA gene [49]. Dimers of MHC-II are formed in the endoplasmic reticulum (ER), then bind with a non-polymorphic invariant chain Ii (CD 74) (Fig. 1b) [50, 51]. Li, also called a pseudo peptide, has a transport function and low affinity for the peptide-binding groove of MHC-II, which can prevent MHCII from binding to premature antigens [52]. MHC II contains targeting motifs that can direct the Ii-MHC-II complex to traffic from the trans-Golgi network (TGN) to the endosomal-lysosomal antigen-processing compartment (MHC-II compartment, MIIC) via clathrin-mediated endocytosis [50]. In the antigen-processing compartment, Li is trimmed gradually by a series of proteases, including cathepsin S, and ultimately SPPL2A, to generate the Ii-associated invariant chain peptide (CLIP). This protects the MHC-II groove before the peptide is bound with MHC-II and removed from the CLIP-MHC-II complex via the enzyme DM (HLA-DM in humans or H2-DM in mice) [53]. DM has a similar structure with MHC-II. It catalyzes peptide acquisition and the dissociation of CLIP in the MIIC through multivesicular bodies (MVB). DM stabilizes MHC-II during peptide interchange and selects for higher binding affinities from the peptide repertoire [50]. After losing CLIP, MHC-II molecules face two possible fates: productively binding with a local peptide and presenting the complex on the cell surface or aggregating and deconstructing the vacant dimers [54]. Although peptide-MHC-II complexes can be generated throughout the endocytic pathway, antigen-processing typically occurs in late endosomal compartments or in lysosomes. These vesicular compartments are enriched with proteolytic enzymes and disulfide reductases. The compartments have sufficiently low pH values to activate these enzymes (Fig. 1c) [34]. Interferon-γ (IFN-γ) induces the expression of the MHC class II transactivator (CIITA), which then converts MHC class II-negative monocytes into MHC class II-presenting functional APCs [55].

### MHC assembly

The receptors on DCs mediate the internalization of antigens into early endosomes, where the pH value is nearly neutral and the activity of antigen-processing enzymes is low [56]. After internalization, the lysosomal enzyme will activate due to the gradual acidification by the ATP-dependent vacuolar proton pump [57]. At first, the longer peptide precursors will bind to MHC-II. The precursors are then trimmed into shorter peptides [50]. The antigen-processing proteases consist of the serine proteases cathepsin A and G (Cat A, G), the aspartic proteases cathepsin D and E (Pepsin family A1A), and the 11 cysteine proteases cathepsins B, C, F, H, K, L, O, S, V, X, and W (Papain family C1A) [58]. Cathepsin S, B, H, and Li are also essential for the degradation of Li from MHC-II [57]. TFEB (transcription factor EB) can also promote lysosome and phagosome acidification and induce protein degradation in DCs [59]. The pH values of the late endosomal and lysosomal-processing compartments reach approximately 3.8–5.0, allowing the endopeptidases (EXPD) to recognize the most susceptible site for subsequent cleavage on the antigens. Then, GILT/IFI30 reduces certain disulfide bonds of the antigens’ secondary structure [54].

### MHC-peptide trafficking in DCs

After proteolytic cleavage, antigens are transferred to nearby MHC-II molecules. In this course, many different “pro-determinants” of antigens are exposed to the acidic vesicular endosomal system [53]. Large pro-determinants may contain more than one MHC-II binding region, but the most suitable, most dominant pro-determinant has the strongest binding affinity for MHC-II. This process is known as competitive capture. Once the pro-determinants are bound, their core residues will be protected by the MHC-II inside the lysosomal compartment. Many antigens contain only one dominant determinant in a haplotype [48]. The ER aminopeptidase has recently been identified to take part in antigen-processing guided by MHC. Recent data showed that a peptide of 51 amino acids did not need to be processed, but it was preferable in the competitive capture process than a peptide of half its size. Compared with the 10-mer cytochrome c peptide, the 23-mer peptide had a 32 times higher binding affinity to MHC-II [60].

Endogenous peptides are generated by proteasomal processing, then imported into the ER where the majority of MHC-I are loaded via the action of the transporter associated with antigen-processing (TAP) (Fig. 1c) [61, 62]. The closed-end of MHC-I molecules only binds to short peptides containing 8–10 amino acids [63]. Before being loaded to MHC-I molecules, the peptide must be trimmed by ER aminopeptidase (ERAP) chaperones, such as calnexin and calreticulin [53]. The specificity of the proteasome, including ERAAP/ERAP1, trypsin, and TAP, can influence epitope generation and transportation to receptive MHC-I molecules [62]. The MHC-I-peptide complex is generally presented to CD8 T cells, which induce the phosphorylation of the ITAM motifs in TCR through a proto-oncogene tyrosine-protein kinase and the Src (SRC) family kinases pathway [54].

Exogenous antigens are usually presented by MHC-II molecules. Before binding to peptides, MHC-II molecules must release CLIP and then generate an open groove for binding [64]. The open groove of MHC-II, containing a 9-amino acid glove cast (3–4 MHC-II anchor residues), tends to bind to longer peptide fragments (> 11 amino acids) [53]. Peptide-MHC-II complexes in DCs leave the antigen-processing compartments and traffic for the plasma membrane, where they can interact with T cells. Microvilli on T cell surfaces act as detectors for these complexes and can continue moving to detect p-MHC. Different peptides are exchanged until the peptide with the highest affinity binds the TCR grooves [65]. Dynamic interactions between APCs and T cells require several hours to several days [66]. The MHC-II peptidome contains high-affinity and low-affinity peptides. IRF4 regulatory CD11b^+^ DC subsets enhance peptide-MHC-II complex formation and present antigens to helper T cells in order to stimulate them [52].

## Antigen presentation

To activate CD8^+^ or CD4^+^ T cells, several signals are needed (Fig. 1d, e): Signal 1: Antigenic peptides bound to MHC-I or MHC-II molecules are presented to CD8^+^ T cells or CD4^+^ T cells, respectively [67]. Signal 2: Appropriate co-stimulatory signaling is delivered through the balance between diverse positive and negative signal s[60]. CD80/CD86 and programmed death-ligand 1 or 2 (PDL1/2) are examples of the positive and negative signals on DC surfaces [68]. Signal 3: T cell stimulatory cytokines are produced by DCs. Examples of such cytokines are proinflammatory interferons (IFNs) and interleukin-12 (IL-12) [69]. These cytokines also stimulate the functional expansion and memory development of CTLs.

### Classic antigen presentation to T cells

The T cell receptor (TCR) or TCR-CD3 complex consists of four subunits—an antigen-binding TCRαβ (or TCRγδ) subunit and three signaling subunits (CD3εδ, CD3εγ, and CD3ζζ)—and initiates antigen-specific immune responses [70]. As they do not contain cytoplasmic signaling motifs, the TCRαβ and TCRγδ subunits cannot trigger intracellular activation signaling pathways upon recognizing antigens on APCs. TCR-mediated signals are transmitted across the cell membrane by CD3 chains, including CD3γ, CD3δ, CD3ε, and CD3ζ. All CD3 chains contain ITAMs in their cytoplasmic domain. CD3ε, CD3γ, and CD3δ each contain one ITAM in their cytoplasmic domain, whereas CD3ζ contains three ITAMs [71]. The antigen presentation process by peptide-MHC to TCRs can be divided into two stages: the transformation of TCR structure from “closed to open” and the phosphorylation activation of the ITAMs of TCR [66]. TCR interaction with distinct peptide-MHC can trigger distinct conformational changes. MHC-I-peptide and MHC-II-peptide complexes on the surface of DCs are presented to TCR complexes on CD8^+^ and CD4^+^ T cells, respectively, which in turn promote T cell activation, proliferation, and differentiation (Fig. 1e, f) [72].

### Cross-presentation and cross-priming

Cross-presentation is the process wherein DCs take up, process, and present extracellular antigens via MHC-I molecules to CD8^+^ T cells. This is also known as cross-priming [73]. Cross-presentation is necessary to activate CD8^+^ T cells and has a considerable effect on immune surveillance in transplants and immune defense in infections. Only DCs can cross-prime for a cytotoxic CD8^+^ T cell response [62]. Particularly, XCR1^+^ DCs are crucial for cross-presentation and communication between CD4^+^ and CD8^+^ T cells in a productive vaccinia virus (VV) infection [74]. Many factors will infect cross-presentation. TLRs can also trigger phagosomal MHC-I transport from the endosomal recycling compartment to facilitate cross-presentation [11]. The absence of FcRn will also impair the cross-presentation of IgG-bound internalized antigens by CD8^−^CD11b^+^ DCs. TFEB can inhibit the DC presentation of exogenous antigens via MHC-I and promote presentation via MHC-II [75].

### Antigen presentation by DC exosomes

**Fig. 2:**
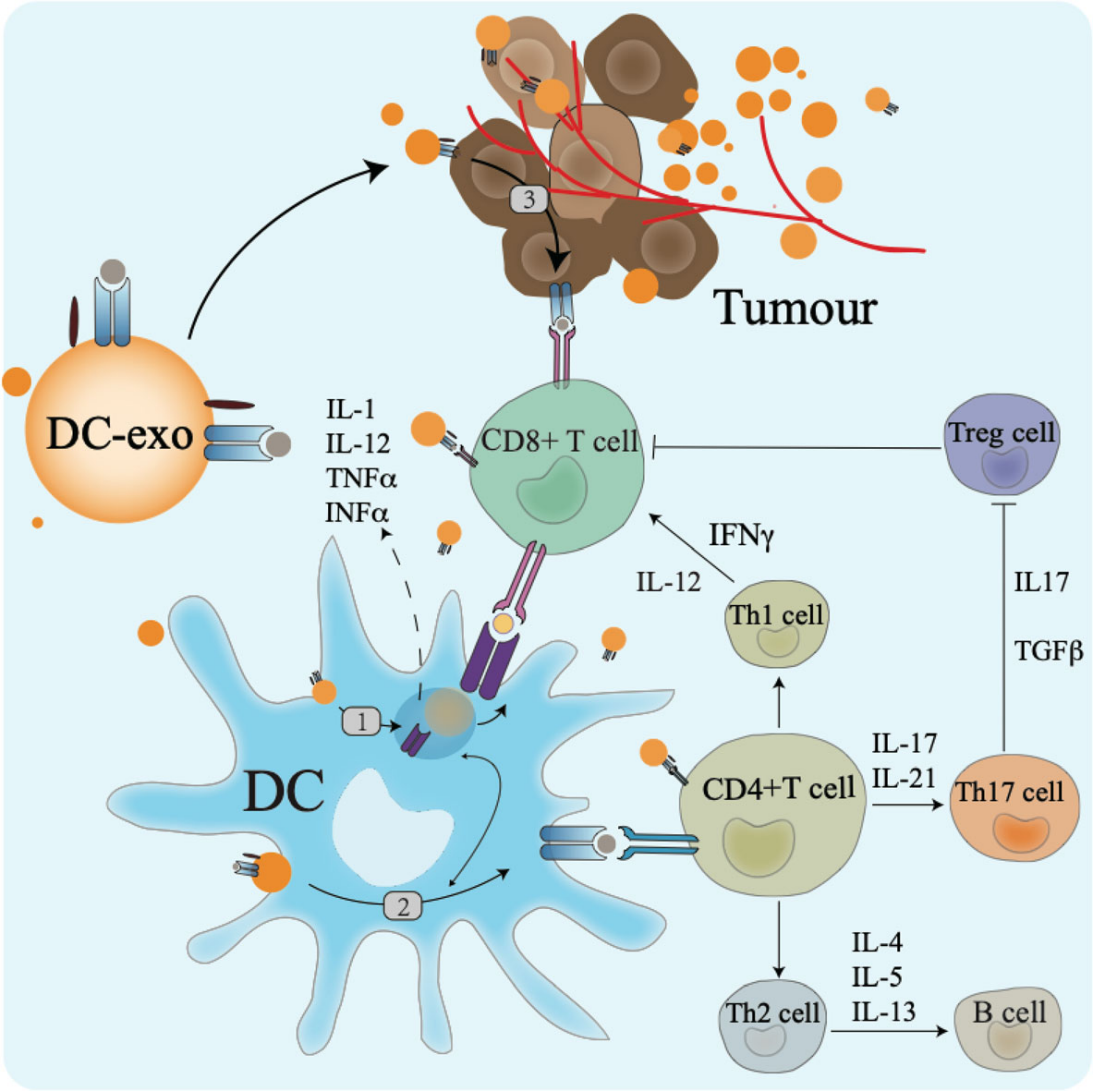
DC exosome-mediated antigen presentation and T cell activation

DC-derived exosomes (Dex) are nanometer-sized membrane vesicles that can migrate to tumors or the spleen and present antigens directly or indirectly to CD4+ and CD8+ T cells, thereby inducing immune responses [76]. Several mechanisms have been proposed on how Dex presents antigens via MHC molecules in order to stimulate T cell responses (Fig. 2).

First, Dex can present antigens to T cells directly, which is thought to be a restimulation of activated T cells [77].

Secondly, a process known as cross-dressing occurs. Simply put, it is Dex-mediated indirect antigen presentation to T cells. After binding to APCs, Dex merges with the acceptor APC surface membrane and transfers its peptide/MHC complexes. Following internalization, the Dex peptide/MHC complexes can be reprocessed via endosomal pathways within the APC. Peptide complexes can then be transported back to the DC’s surface for presentation to T cells.

Thirdly, Dex can be internalized by tumor cells and convert tumor cells into stronger immunologic targets for effector immune cells [77]**.** The mature Dex can activate immature DCs and T cells in vitro [78]. Rao et al. reported that DCs pulsed with exosomes from the human hepatocellular carcinoma HepG2 cell line could elicit a stronger antigen-specific CTL response than cell lysates did in vitro and in vivo [79]. DCs can also secrete extracellular vesicles (EVs) of different sizes [76]. Large EVs (lEVs) secreted by immature DCs induce Th2 cytokine secretion (IL-4); small EVs (sEVs) induce Th1 cytokine secretion. Upon DC maturation, all EVs induce Th1 cytokine secretion [76, 80].

## Immune responses meditated by DCs

### DCs enable CD4^+^ T cells to activate B and CD8^+^ T cells

According to their patterns of cytokine production, transcription factor expression, and cell surface marker expression, CD4^+^ T helper cells are currently subdivided into multiple lineages, encompassing at least Th1, Th2, Th17, and follicular T helper (Tfh) (Fig. 2). CD40, a co-stimulatory molecule glycoprotein with 277 amino acids also known as TNFRSF5, was originally identified as a receptor on B cells and was later found to be expressed in various other immune effector cells. T follicular helper cells, a subgroup of T cells, mediate important cell-cell interactions with B cells that occur within the follicles of secondary lymphoid organs. These T cells also stimulate and govern B cells to produce antibodies. The interaction of CD40 on DCs in addition to CD40L on T cells leads to DC activation, enabling DCs to prime T cells and induce the upregulation of co-stimulatory molecules, adhesion molecules, and the Th1-polarizing cytokine IL-12 in both mouse and human DCs [81]. Notably, the IL-12 produced after the interaction of CD40 with CD40L plays a decisive role in determining the type of CD4^+^ T cell immunity [69]. IL-12 polarizes the differentiation of naive CD4^+^ T cells into Th1 cells [82]. Th1 and Th2 cells, in turn, secrete interleukin IL-2, IFNγ, IL-4, IL-5, and IL-13, respectively, to promote CD8^+^ T and B cell responses [64]. Th1 cells express the defining T-box transcription factor TBX21 (T-bet), express chemokine receptors such as CXC-chemokine receptor 3 (CXCR3) and CC-chemokine receptor 5 (CCR5), and secrete IFNγ. Moreover, many CD4^+^ T cells in the atherosclerotic plaque express other Th1-associated proinflammatory cytokines in addition to IFNγ, such as IL-2, IL-3, tumor necrosis factor (TNF), and lymphotoxin, which can all activate macrophages, T cells, and other plaque cells, accelerating the inflammatory response [83]. The main Th2 cell cytokine is IL-4. IL-4 binds to the IL-4 receptor on T cells and activates signal transducer and activator of transcription 6 (STAT6), leading to the expression of the transcription factor GATA3, the master regulator of Th2 cell differentiation. In mouse atherosclerotic plaques, a substantial proportion of T cells express transcripts for Th2 cell-associated cytokines, such as IL-4, IL-5, IL-10, and IL-13 [84].

Recent studies have revealed that primary tumors can induce B cell accumulation into draining lymph nodes (DLN), possibly through signaling mediated by the phosphorylated proteins EGFR, VAV2, P130, CHK2, and CLDN3 in DLN [85–88]. When B cells accumulated in the DLN, they increased the expression of cell cycle related genes Cdc25c, Bub1, Ttk, and Cdk1, and migration-related genes Vcam1, Arhgap5, Cxcr3, and Ccr2. They also secreted chemotactic molecules. In the meantime, these B cells selectively promoted cancer cell lymph node metastasis by producing pathogenic IgG that targeted the glycosylated membrane protein HSPA4 of cancer cells. HSPA4 targeting IgG activated the HSPA4-binding protein ITGB5 and the downstream Src/NF-κB pathway in cancer cells to promote CXCR4/SDF1α-axis-mediated cancer metastasis [85, 87, 88].

### DCs mediate immune memory

Immune memory is a vital mechanism of myeloid cell plasticity. It occurs in response to environmental stimuli and alters subsequent immune responses [89]. Two types of immunological imprinting can be distinguished: training and tolerance. These imprinting processes are epigenetically mediated and enhance or suppress subsequent inflammation, respectively [89]. DCs can also mediate immune memory via group 2 innate lymphoid cells (ILC2) [90]. Memory Th2 cells are essential for the recall response and subsequent type-2-cytokine-driven inflammation [90, 91]. Halim et al. reported that ILC2 is critical in memory Th2 cell immune response [90]. Activated ILC2 can secrete IL-13 to stimulate IRF4^+^CD11b^+^CD103^−^DCs, generating CCL17 and recruiting CCR4^+^ memory Th2 cells [90]. To generate a long-term vaccinal anti-tumor response, many researchers are investigating the conversion of effector T cells into memory T cells. The desired anti-tumor antibodies should be optimized against cytotoxic effects and should be involved in motivating a long-lasting anti-tumor cellular immune response [92]. DiLillo et al. demonstrated that both hFcγRIIIA expressed on macrophages and hFcγRIIA expressed on human DCs (Table 1) generated a potent long-term vaccinal anti-tumor T cell response upon ADCC-mediated tumor clearance in a FcγR-humanized murine lymphoma model. Zhang et al. reported that CD45^+^RALDH^+^ DCs controlled volume expansion and maintenance in the secondary lymphoid organs of germ-free mice [93]. Many factors enhance DC active stages. For example, Zanoni et al. found that microbial products and self-encoded oxidized phospholipids (ox-PAPC) can make DCs hyperactive via a caspase-11 enzyme that bound to ox-PAPC and a bacterial lipopolysaccharide (LPS). Hyperactive DCs are longevous and can convert effector T cells into memory T cells [94].

### DCs’ effects on Tc1 and Treg cells

The cardinal features of natural or therapy-induced immuno-surveillance are CD8^+^ cytotoxic T lymphocytes (Tc1 cells), which can specifically recognize antigens and produce a particular interferon-γ (IFN-γ)-centered cytokine pattern [95, 96]. For major human malignancies, the abundance of Tc1 cells in tumors has a positive prognostic impact. It is activated by IL-12 and CCR7-mediated CD103^+^/CD141^+^ DCs [95]. CCR7 loss in DCs leads to deficient lymph node T cell activation and will increase tumor outgrowth [96]. CCR7 expression levels in human tumors correlated positively with signatures of CD141^+^ DCs and intra-tumor T cells, as well as better clinical outcomes [96].

DCs present peptide-MHC to TCR and generate IL-2 to promote the development of antigen-specific Treg cells for immune suppression. High levels of type I IFNs will feedback suppress Treg cell expansion [97, 98]. When type I IFNs wane, Treg cells increase the expression of IL-10 to suppress the maturation state of DCs and limit their production of proinflammatory cytokines [99]. Low levels of proinflammatory signals allow for the continued maturation of effector CD8^+^ T cells into functional memory CD8^+^ T cells [100]. Indoleamine 2, 3-dioxygenases (IDO1) expressed in DCs can deplete tryptophan and increase kynurenine, which in turn activates Treg cells and exerts important immunosuppressive functions [101].

### DC and NK cells crosstalk

The reciprocally activating crosstalk between DCs and NK cells plays a pivotal role in the innate immune response against cancer and infections [102]. DCs recruit NK cells to the draining lymph nodes and interact with them in a CXCR3-dependent fashion. DCs and NK cells interact through a “touch and go” mode lasting from 300 s to 4 h [103]. The interaction induces DCs to produce cytokines IL-12, IL-18, IL-27, type I IFNs, IL-15, and prostaglandin E2 (PGE2), leading to the proliferation of NK cells, the expression of the activation marker CD69, and the release of the effector molecule IFN-r [102]. During viral infection, DCs can be recruited to the infection site through the type I interferon mediated production of the chemokine CCL2. The recruited DCs are then activated via SIGN-R1 triggers to produce the chemokines CCL5, CXCL9, and CXCL10, which recruit NK and T cells to the infected site to kill the viruses. As a negative feedback molecule, IL-10, produced by the interacting cells, was able to limit this process [103–105]. Activated NK cells may leave the lymph node, infiltrate tumors, and kill cancer cells in tumors. In contrast to CD21^+^ NK cells, the activated CD2^+^ NK cell subset produces IFN-γ. This induces DC maturation and stimulates T cell responses. This also kills autologous immature DCs through the CD94/NKG2A inhibitory NK receptor [102].

## DCs in tumor immunity and immunotherapy

Cancer cells often escape from immune surveillance and sometimes show relative resistance to chemotherapeutic drugs. Tumors contain heterogeneous cancer cells, including tumor stem cells [106, 107], which interact with stromal cells and immune cells in the tumor microenvironment [108]. DCs, as crucial APCs, mediate tumor immunity via the activation of CD8^+^ and CD4^+^ T cells (Fig. 1). In addition, exosomes expressing CD47 to protect themselves from phagocytosis by monocytes and macrophages have been used in tumor immunotherapy and have impressive outcomes [109]. DCs have been used for tumor immunotherapy in various kinds of preclinical and clinical studies. We categorized the included studies (Table 3), which may reflect clinical importance. We also note that viruses have been used for tumor virotherapy and immunotherapy [127–129]. Prospective studies need to be warranted to investigate the clinical benefits of cancer immunotherapy in combination with virotherapy through DC immunotherapy.

**Table 3:** Clinical trials of dendritic cells in cancer immunotherapy

DC	Tumor type	Combination therapy	Route, dose	Comparison (Medication group and control group)	Efficacy (partial response, PR; complete response, CR; overall survival, OS; progression-free survival, PFS)	Safety (grade III and IV adverse events)	Phase (I, II, III, n)	Trial registration	Ref.
CMV pp65 RNA-loaded DCs	Glioblastoma	CMV pp65-specific T cells		17 patients were randomized to receive CMV pp65-specific T cells with CMV-DC vaccination or saline	Increased in polyfunctional CMV-specific CD8+ T cells, correlated with overall survival		I (17)	NCT00693095	[110]
CMV pp65 mRNA pulsed DCs	Glioblastoma	DI-TMZ, GMCSF	DI-TMZ (100 mg/m2/d × 21 days per cycle), at least three DC vaccines, GM-CSF on day 23 ± 1 of each cycle.	Single arm	Median PFS and OS were 25.3 months and 41.1 months		I (11)	NCT00639639	[111]
hTERT-DCs	Acute myeloid leukemia		1 × 10^7^ cells, 6 weekly injections, 6 biweekly injections	Single arm	Median follow-up of 52 months, 58% of patients in CR		II (36)	NCT00510133	[112]
DCs electroporated with Wilms' tumor 1 (WT1) mRNA	Acute myeloid leukemia		Intradermal injection of 0.5 × 1e6 WT1/DCs on the back of the patient	Single arm: 30 patients with AML at very high risk of relapse	5 year OS was higher in responders than in nonresponder patients. Age ≤ 65 and > 65, 5 year OS was 69.2% and 30.8%		II (30)	NCT00965224	[113]
HER2 peptide-pulsed DC1s	HER^pos^ breast cancer			Patients were randomized for different injection routes	CR for ductal carcinoma in situ, invasive breast cancer patients was 28.6% and 8.3%	Well tolerated	I (54)	NCT02061332	[114]
Autologous tumor lysate plus DC	Metastatic colorectal cancer (mCRC)			52 patients were randomized to DC + best supportive care (BSC) vs. BSC	Median OS was 6.2 months in DC+BSC versus 4.7 months in BSC		III (52)	NCT01413295	[115]
DCs pulsed with killed PCa cells	Prostate cancer	Chemotherapy	Metronomic cyclophosphamide 50 mg p.o., DCVAC/PCa 1 × 10^7^ dendritic cells per dose injected	Single arm: progressive metastatic castration-resistant prostate cancer	The median OS was 19 months		I/II (25)	CT 2009-017295-24	[116]
Autologous DCs pulsed with allogeneic tumor cell lysate	Mesothelioma		First, second and third cohort received 10, 25, 50 million monocyte-derived DCs per vaccination	9 patients divided over three different dose cohorts	Median PFS was 8.8 months	No dose limit	I (9)	NCT02395679	[117]
Autologous activated DCs	Lung cancer	Activated killer T cell AKT, chemoth., EGFR-TKI		Group A, immunotherapy; group B, chemotherapy.	The 2- and 5-year OS rates were 96.0 and 69.4% in group A and 64.7 and 45.1% in group B		III (103)	UMIN000007525	[118]
Activated DCs	Diverse solid tumors		Intratumorally injected at 2, 6, 15 million aDCs	Single arm	OS and TNFα levels increased		I (39)	NCT01882946	[119]
Tumor antigen-pulsed DCs	Hepatocellular carcinoma		Dendritic cell vaccines injected subcutaneously near to inguinal lymph nodes.	Single arm: patients with no viable tumor after primary treatments were included	9 patients had no tumor recurrence up to 24 weeks	Primary treatment for HCC.	I/IIa (9)	KCT0000427	[120]
Peptide-pulsed DCs	Pancreatic cancer (PC)	Toll-like receptor (TLR)-3 agonist poly-ICLC	Peptide-pulsed DC vaccines every 2 weeks. Concurrent intramuscular administration of Poly-ICLC	Single arm: 9 patients with metastatic PC, and 3 patients with locally advanced unresectable PC	Median overall survival was 7.7 months. One patient survived for 28 months		I (11)	NCT01410968	[121]
mRNA Electroporated DCs	Advanced melanoma	TriMixDC-MEL plus ipilimumab	Intradermally and intravenously plus ipilimumab every 3 weeks, then every 12 weeks	Single arm	6-month disease control rate was 51%, the overall tumor response rate was 38%		II (39)	NCT01302496	[122]
Ad-CCL21 Gene-Modified DCs	NSCLC	None	2 vaccinations by intratumoral injections	Single arm: stage IIIB/IV NSCLC	4/16 patients had stable disease at day 56. Median survival was 3.9 months		I (16)	NCT01574222	[123]
Autologous tumor lysate pulsed with DCs	Bone and soft tissue sarcoma		6 weekly DC injections into the inguinal or axillary region.	Single arm: metastatic or recurrent sarcomas	The 3-year overall and progression-free survival rates were 42.3% and 2.9%		I/II (37)		[124]
Autologous tumor cell pulsed Dcs (VAX-DC/MM)	Multiple myeloma	Monocyte-derived immature DCs	Intradermal VAX-DC/MM injection of 10 × 1e6 cells every week for 4 weeks	Single arm: relapsed or refractory MM	Most patients (77.8%) who received 10 × 1e6 cells showed an immunological response		I (12)	NCT02248402	[125]
None	High-risk stage III/IV melanoma	GM-CSF, multiepitope melanoma peptide		A multicenter intergroup randomized placebo-controlled trial	11.3% vs. 27.1% patients developed peptide-specific CD8+ T cell responses.		III (815)	NCT01989572	[126]

### DC vaccines showed great potential for tumor immunotherapy

Tumor-specific antigens are being used to stimulate DCs. These antigens include cancer-testis or cancer-germline antigens, abnormally expressed fetal antigens, mutated antigens, overexpressed antigens, differentiation antigens, and viral antigens [130]. Culturing patient tumor cells with allogeneic-IgG-loaded DCs induced vigorous patient T cell responses to autologous tumor antigens, shedding light on this technique as a new potent method for tumor immunotherapy [131]. Personalized DC vaccines have induced T cell immunity, which targets private somatic neoantigens in certain melanoma patients and may become clinically feasible soon [132]. Personalized DC vaccines can be generated by the co-culture of autologous DCs with oxidized autologous whole tumor cell lysate (OCDC) that has been shown to significantly prolong patient survival [133]. Also, allogenic mature DCs have been made to fuse with inactive gastric cancer cells (MGC803) and cytokine-induced killing cells (CIKs), facilitating efficient, targeted immunotherapy against gastric cancer [134]. It has been found that fusion cells (FCs) in addition to CIKs can trigger tumor-specific CTLs and inhibit tumor growth in vivo. FCs can act as efficient vehicles to deliver tumor antigens systemically by activating CTL and triggering an anti-tumor immune response [134]. Mitchell and his colleagues found that a tetanus/diphtheria (Td) toxoid can induce CCL3 expression and facilitate DC migration. They deployed a DC vaccine pulsed with glioblastoma specific antigen cytomegalovirus phosphoprotein 65 (pp65), which was able to enhance anti-tumor effects [135].

### DCs in combination tumor immunotherapy

Effective tumor immunotherapy requires four parts as follows: a tumor antigen targeting antibody, recombinant interleukin-2 with an extended half-life, anti-PD1, and a powerful T cell vaccine [136]. These combined therapies promote immune cell infiltration and inflammatory cytokine production. Curative tumor regression is mediated mainly by CD8^+^ T cells and cross-presenting DCs, suggesting that effective treatment engages innate and adaptive immune responses to eradicate large tumors [136]. The identification of human cancer-specific antigens has led to the development of antigen-specific immunotherapy in cancer. CD47 is a transmembrane glycoprotein widely expressed on the surface of cancer cells [73], which, embedded on exosomes, limits their clearance by circulating monocytes [109]. It transmits an inhibitory signal through its receptor—the signal regulatory protein alpha (SIRPα) on DCs. This signal blunts antibody effector functions as an antiphagocytic ligand exploited by tumor cells [137]. The interference with CD47–SIRPα interaction synergized with tumor-specific monoclonal antibodies enhanced macrophage-mediated antibody-dependent cellular phagocytosis (ADCP), leading to the elimination of human tumor xenografts in mice [137]. Exosomes harboring SIRPα variants (SIRPα-exosomes) were sufficient to induce augmented tumor phagocytosis, resulting in a prime, effective anti-tumor T cell response. This suggests that a superlative exosome-based platform has broad potential to maximize the therapeutic efficacy of membrane-associated protein therapeutics [138]. Interestingly, near-infrared photoimmunotherapy (NIR-PIT) is a localized molecular cancer therapy combining a photosensitizer-conjugated mAb and light energy. CD47-targeted NIR-PIT increases direct cancer cell death and phagocytosis, resulting in inhibited tumor growth and improved survival in a model of human bladder cancer [139]. A novel CD47-targeting fusion protein, termed SIRPαD1-Fc, was generated and found to increase the phagocytic and cytotoxic activities of macrophages against non-small cell lung cancer (NSCLC) cells [140]. Targeting both CD47 and autophagy in NSCLC xenograft models elicited enhanced anti-tumor effects, with the recruitment of macrophages, activated caspase-3, and overproduction of ROS at the tumor site [140, 141].

DCs and cancer cells express PDL1 on their cell surface, which represses T cell activation [142]. Specific antibodies that block immune checkpoint molecules, such as the cytotoxic T lymphocyte antigen 4 (CTLA4), PDL1, and PD1 are currently licensed as therapies for various types of cancers [100, 143]. Mezzadra et al. found that CMTM4 can help CMTM6, a type-3 transmembrane protein, to reduce PDL1 ubiquitination and increase its protein half-life, enhancing the ability of PDL1-expression in tumor cells to inhibit T cells [144]. Arming Abs with IFN-β is more potent than the first generation of Abs in controlling Ab-resistant tumors [145]. Yang et al. found that DCs were the major cell type responding directly to anti-EGFR-IFN-β treatment by increasing antigen cross-presentation. Combined therapy with anti-EGFR-IFN-β and PDL1 blocking completely eradicated established tumors [145]. In addition, Overacre-Delgoffe et al. found that neuropilin-1 (Nrp1)-deficient Tregs induced IFN-γ, which made intratumoral Tregs fragile and boosted anti-PD1 therapy [146]. The TLR7 antagonist Loxoribin inhibited tumor growth in xenograft models of colon cancer and lung cancer by promoting CD4^+^ T cell proliferation, reversing CD4^+^CD25^+^ Treg-mediated suppression via DCs [13, 147]. DC cross-presentation can also reactivate CTL and block PDL1 induced by IFN-γ [68]. T cell therapy needs CD40-CD40L to activate the tumor necrosis factor (TNF) and DCs to produce nitric oxide synthase 2 (NOS2) [60].

### DCs promote tumor immunotherapy by suppressing Treg cells

DC-based cancer immunotherapy is a promising approach, but Treg cells in the tumor microenvironment are the biggest barrier for effective tumor immunity. Treg cells and DCs in the tumor microenvironment can mutually suppress each other [148]. DCs can suppress Treg cells but activate effector T (Teff) cells to enhance tumor immunity by inhibiting the p38 MAPK pathway through the DC cell surface molecule OX40L [149]. Additionally, OX40 co-stimulation by SB202190-treated mDCs (mSBDCs) inhibits the conversion of Teffs to Tregs [149]. In the tumor microenvironment, tumor-associated DCs can produce reactive oxygen species (ROS), which cause lipid peroxidation/degradation and tumor suppression. Meanwhile, the accumulation of unfolded proteins in the ER can also cause ER stress, which in turn enhances unfolded protein response (UPR), resulting in the reduced DC expression of MHC-I molecules and an impaired anti-tumor T cell response. This indicates that ER stress in DCs suppresses tumor immunity via MHC-I expression reduction [150, 151].

### Clinical trials of DC-based tumor immunotherapy

Clinical trials of DC-related cancer immunotherapy show promising results (Table 3). These trials may be classified into DC vaccines and other DC-related trials. DC vaccines involve DCs that recognize various kinds of tumor-specific antigens or whole tumor lysates, as well as cytokine activated DCs. Other DC-related trials may not use DCs directly, but DCs are involved in their therapeutic mechanisms.

DC vaccines have been tested in multiple clinical trials to target many tumor-specific or tumor-associated antigens, including CMV pp65, telomerase, Her2, Wilms’ tumor 1, and so on. Two stage I clinical pilot trials used vaccination with CMV pp65 mRNA-loaded DCs in patients with glioblastoma (GBM). Patients who received this vaccination experienced an increase in the overall frequencies of IFNγ^+^, TNFα^+^, CCL3^+^ polyfunctional, and CMV-specific CD8^+^ T cells, as well as long-term progression-free survival alongside overall survival [110, 111]. Telomerase activity in leukemic blasts is frequently increased among patients with high-risk acute myeloid leukemia (AML). In a stage II clinical study, the researchers found that human telomerase reverse transcriptase (hTERT)-expressing autologous DCs (hTERT-DCs) were feasible. Vaccination with hTERT-DCs appeared to be safe and may be associated with favorable recurrence-free survival in adult patients with AML [112]. DCs electroporated with Wilms’ tumor 1 (WT1) messenger RNA (mRNA) were found to be an effective strategy to prevent or delay AML relapse after standard chemotherapy with the induction of WT1-specific CD8^+^ T cell response in a stage II clinical trial [113]. In the clinical trial anti-HER2, DC1s vaccination was a safe and immunogenic treatment to induce tumor-specific T cell responses in HER2^pos^ breast cancer patients [114]. In another trial, Wilms’ tumor 1 peptide-loaded DCs and OK-432 adjuvant combined with conventional chemotherapy was shown to be safe and feasible for patients with an advanced stage of head and neck squamous cell carcinoma (HNSCC) [152].

An autologous tumor lysate DC vaccine was shown to have T cell stimulatory capacity. It generated a tumor-specific immune response and benefitted the overall survival of metastatic colorectal cancer patients in a stage III clinical trial [115]. Autologous mature DCs pulsed with killed LNCaP prostate cancer cells (DCVAC/PCa) in addition to concomitant chemotherapy did not preclude the induction of specific anti-tumor cytotoxic T cells in a I/II clinical trial study [116]. Some autologous DCs generated ex vivo pulsed with tumor antigens showed limited promise in the treatment of patients with advanced cancers. In a stage I clinical trial, autologous DCs pulsed with allogeneic tumor cell lysate demonstrated that DC immunotherapy with allogeneic tumor lysate can be safe and feasible in humans [117]. The adoptive transfer of autologous activated killer T cells and DCs (AKT-DC) in a stage III clinical trial elevated the CD8^+^/CD4^+^ T cell ratio in survivors of patients with non-small cell lung cancer [118]. Intratumoral activated DC injections in a stage I clinical trial increased the production of specific cytokines and prolonged survival as well [119].

Furthermore, in a stage I/II clinical trial, it was shown that pre-conditioning the vaccine site with a potent recall antigen such as the tetanus/diphtheria (Td) toxoid significantly improved lymph node homing and the efficacy of tumor antigen-specific DCs [120, 130]. In one study, patients with glioblastoma were pre-conditioned with either mature DCs or Td before a vaccination with cytomegalovirus phosphoprotein 65 (pp65) mRNA pulsed DCs. The results indicated that this may represent a viable strategy to improve anti-tumor immunotherapy [130].

Other DC-related trials include the use of DCs in conjunction with the toll-like receptor (TLR)-3 agonist poly-ICLC against metastatic or locally advanced unresectable pancreatic cancer. Results showed an increased tumor-specific T cell population [121]. Additionally, in a stage II clinical trial, autologous monocyte-derived mRNA electroporated DCs (TriMixDC-MEL) alongside ipilimumab usage resulted in durable tumor responses in melanoma patients [122]. In a stage I clinical trial, DCs were transduced with an adenoviral (Ad) vector expressing the CCL21 gene (Ad-CCL21-DC), which induced systemic tumor antigen-specific immune responses, enhanced tumor CD8^+^ T cell infiltration, and increased tumor PDL1 expression [123].

## Conclusions and perspectives

DCs are crucial sentinel cells. Educating naive T cells for adaptive immune responses, DCs recognize antigens, process antigens into small bioactive peptides, and form specific MHC-peptides complexes before presenting antigens to T cells. DCs are not only able to activate T cells, but they also maintain a balance among immune activation, suppression, and memorization. Thus, DCs, the mentors of T cells, are a key player in immune defense, surveillance, and homeostasis. Furthermore, accumulating evidence indicates that DCs are a key player in tumor immunity. DC-based tumor immunotherapy has been shown to be highly effective in preclinical studies and clinical trials. DCs can specifically recognize, process, and present diverse and heterogeneous cancer antigens, as well as activate T cells specifically to overcome drug resistance caused by cancer cell heterogeneity. DC-based tumor immunotherapy has shown great potential in a wide variety of tumors.

The increasing applications of new technologies and hypotheses to DC research will likely reveal more insights in our fundamental understanding of DC biology. Future works can easily promote the development of new strategies for DC-based tumor immunotherapy, and we believe that DC-based tumor immunotherapy holds great promise for a cure to cancer in future.

## Data Availability

Not applicable.

## Abbreviations

DCs: Dendritic cells
APCs: Antigen presenting cells
lncRNAs: Long non-coding RNAs
HSCs: Hematopoietic stem cells
MHC: Major histocompatibility complex
CTL: Cytotoxic T lymphocyte
PAMPs: Pathogen-associated molecular patterns
DAMPs: Danger-associated molecular patterns
PRRs: Pattern recognition receptors
CLRs: C-type lectins receptors
GPCRs: FPRs are G protein-coupled receptors
NLRP3: Pyrin domain-containing 3
ITAM: Immune receptor tyrosine-based activation motif
ITIM: Immune receptor tyrosine-based inhibitory motif
TRIM21: Tripartite motif-containing protein 21
TGN: Trans-Golgi network
BRS: Basic residue-rich sequence
VV: Vaccinia virus
HCC: Hepatocellular carcinoma
IDO1: Indoleamine 2, 3-dioxygenases
ER: Endoplasmic reticulum
OCDC: Co-culture of autologous DCs with oxidized autologous whole tumor cell lysate
ADCP: Antibody-dependent cellular phagocytosis
NIR-PIT: Near-infrared photoimmunotherapy
NSCLC: Non-small cell lung cancer
HNSCC: Head and neck squamous cell carcinoma
